# Soil redox potential and plant species identity likely affect root-associated fungi in salt marshes

**DOI:** 10.1007/s00572-026-01310-2

**Published:** 2026-09-24

**Authors:** Andrea-Carolin Menzel, Lina Kleemann, Petra Bukovská, Jason N. Woodhouse, Peter Mueller, Kai Jensen, Jan Jansa

**Affiliations:** 1 Institute of Plant Science and Microbiology, University of Hamburg, Hamburg, 22609 Germany; 2 Institute of Microbiology, Czech Academy of Sciences, Prague, 14200 Czech Republic; 3 Department of Biology, University of Hamburg, Hamburg, 20148 Germany; 4 Leibniz-Institute of Freshwater Ecology and Inland Fisheries, Stechlin, 16775 Germany; 5 Institute for Environmental Sciences, RPTU Kaiserslautern-Landau, Landau, 76829 Germany

**Keywords:** Arbuscular mycorrhizal fungi, Environmental stress, Marine-terrestrial ecotone, Marsh zones, Root-associated fungi, Soil oxygen availability

## Abstract

Root-associated fungi (RAF) are important for plant performance because they form beneficial, neutral, or pathogenic relationships with their hosts, thus regulating nutrient uptake, stress tolerance, and overall plant fitness. At the transition from marine to terrestrial ecosystems, salt marshes are shaped by strong environmental gradients and provide vital ecosystem services such as climate regulation and coastal protection, yet RAF remain poorly understood in these systems. Therefore, we examined the community structure and abundance along this ecotone of RAF in general, and arbuscular mycorrhizal fungi (AMF) in particular, using microscopy to assess AMF abundance in plant roots, high-throughput sequencing to determine fungal community composition, and measured abiotic soil conditions. The observed shift in fungal community from marine-dominated zones at low elevations to those at the terrestrial end at higher elevations corresponded to a pronounced increase of AMF, from 0.04 ± 0.17% to 14.05 ± 8.61% in arbuscular colonization of roots and from 0.85% ± 2.55% to 19.75% ± 11.77% in relative (root DNA-derived) amplicon abundance of Glomeromycota. It mainly correlated to an increase of the soil redox potential, a proxy for oxygen availability (RDA, R^2^ = 0.05, *p* = 0.004). Within similar environmental conditions of a zone, host species identity became an additional strong biotic filter for the RAF community. Our findings offer new insights into the distribution of fungal communities along the marine-terrestrial ecotone and highlight the need for further research to understand mechanisms of their community assembly, their function and responses to ongoing global change.

**Supplementary Information:**

The online version contains supplementary material available at https://doi.org/10.1007/s00572-026-01310-2.

## Introduction

Root-associated fungi (RAF) are fundamental components of vegetated ecosystems, forming close associations with virtually all vascular plants. Through these relationships, the fungi facilitate the uptake of nutrients from soil to plants, improve the plant-water relations, and enhance resistance of plants to a range of biotic and abiotic stressors (Rillig et al. 2015; van der Heijden et al. 2015). Beyond single plant-level benefits, RAF play critical roles in shaping host community composition (van der Heijden et al. 1998). These host-specific communities inside roots (including pathogens and mutualists) drive plant-soil feedback that mediate interspecific competition and species coexistence in plant communities (Kuang et al. 2021). Because positive interactions between plants and microbes are often more prevalent in stressful environmental conditions (Bertness and Callaway 1994), RAF might play a particularly important role in coastal wetlands. In these highly dynamic systems, salinity, inundations, sediment compaction, and associated shifts in oxygen availability collectively impose strong physiological constraints on plant metabolism and microbial activity both in the plants and in the surrounding soil (Veldhuis et al. 2019; Rinke et al. 2022).

However, global reviews reveal that microbial research in coastal wetlands has so far disproportionally focused on bacterial communities, while archaea and fungi have received comparatively less attention (Farrer et al. 2022; Birnbaum et al. 2025). To predict the effects of fungal activity for ecosystem services, restoration, and climate resilience of coastal ecosystems, these knowledge gaps must be addressed. In recent years, coastal ecosystems are increasingly recognized for their important ecosystem services like sediment stabilization, nutrient cycling, carbon sequestration, and biodiversity conservation (Temmink et al. 2022; Liang et al. 2023; Liu et al. 2024). Below-ground plant-microbe interactions may mediate these services directly or indirectly. This seems particularly relevant for RAF, with their functional range from mutualistic to neutral to pathogenic for plants (Lumibao et al. 2024). Yet, despite their recognized ecological importance in upland terrestrial systems, the diversity, distribution, and functional roles of RAF in wetlands remain understudied (Birnbaum et al. 2025). With this study, we aim to address this knowledge gap by exploring RAF community composition and occurrence along the marine-terrestrial ecotone in coastal wetlands at the Germany North Sea Coast.

There is growing evidence that RAF influence plant community structure and support plant growth, establishment, and stress tolerance in wetlands (Wolfe et al. 2007; Clay et al. 2016; Shearin et al. 2018). At the same time, stress-induced changes in root exudation and oxygen availability may favor opportunistic or pathogenic behavior of some RAF. Consequently, root infection by pathogenic taxa increases, impairing root growth and nutrient uptake, ultimately weakening overall plant performance (Martínez-Arias et al. 2022; Grüterich et al. 2024). Additionally, host species identities themselves and their respective traits can mediate these responses. Specifically, differences in root architecture, exudation patterns, oxygen transport capacity, and associated root oxygen loss, as well as nutrient acquisition strategy, may directly affect the success of fungal growth and establishment (Li et al. 2022; Negre Rodríguez et al. 2025). Depending on environmental conditions and host status, these findings suggest that RAF in wetland plants can have a dual role, enhancing stress tolerance or contributing to stress-induced damage.

As characteristic ecosystems of the marine-terrestrial gradient, salt marshes provide a powerful model system to disentangle these interacting drivers. Along elevational gradients of only a few decimeters, strong shifts in inundation frequency, soil salinity, moisture and aeration, and soil pH occur simultaneously (Suchrow et al. 2015; Bakker et al. 2016). These co-varying abiotic factors may jointly structure RAF communities through environmental filtering, defining the range of taxa capable of persisting under different stress regimes (Goldmann et al. 2016; Germain et al. 2018). Moreover, plant identity can further shape which fungi are able to colonize and grow inside roots, reflecting hierarchical community assembly processes described for host-associated microbiomes across various ecosystems (e.g. Botnen et al. 2020; Li et al. 2022; Liang et al. 2023).

Salt marshes along the German North Sea coast show a clear vegetation zonation depending on flooding frequency and elevation (Bakker et al. 2016; Elschot et al. 2024). These salt marsh zones are often dominated by single or few plant species (Wanner et al. 2014) and species distribution strongly depends on the elevational gradient of a total extent of approximately 3 m (Suchrow et al. 2015): *Salicornia europaea* and *Spartina anglica* grow in the marine-dominated pioneer zone (PIO), which is at the lowest end of the gradient, therefore flooded twice daily, and characterized by highly saline and anoxic soils. The low marsh (LM) at mid-elevation is flooded during spring tides around new and full moon, usually has more aerated but still highly saline soils, and supports *Atriplex portulacoides* and *Puccinellia maritima* along with about ten other plant species. The high marsh (HM) at the highest elevation represents the terrestrial end of the ecotone, which is flooded mainly during winter storm surges, but is often subject to severe drought periods in the summer. Here, *Elymus athericus* and *Festuca rubra* are major components of the vegetation. Within these systems, plant-specific root traits, related to root oxygen loss, aerenchyma formation, and exudation patterns, affect the composition and activity of belowground microbial communities (Wang et al. 2017; Hernández et al. 2020; Liu et al. 2024). Generally, studies in coastal wetlands show that arbuscular mycorrhizal fungi (AMF) abundance declines with more frequent inundation (Hildebrandt et al. 2001; Carvalho et al. 2004; Wilde et al. 2009). However, seasonal, site-specific, and host-related variation indicate more complex dynamics (Daleo et al. 2008; Druva-Lusite and Ievinsh 2010). So far, most existing research has focused on soils or spores, often reporting AMF genera such as *Glomus* and *Acaulospora* (Tsiknia et al. 2021; Ma et al. 2024), while little is known about broader community composition of fungi living inside the roots alongside AMF. As a result, the community and abundance of RAF including beneficial mycorrhizal associations and pathogenic and endophytic species (the latter referring to taxa causing no strong antibiosis), remain poorly characterized along the marine-terrestrial ecotone in general, and in salt marshes specifically.

By targeting both RAF in general and AMF in particular, this study aims to advance our understanding on effects of environmental conditions and plant species identity on fungal communities using Wadden Sea salt marshes as a model system. We hypothesize that (H1) RAF communities are structured along the elevational gradient in the marine-terrestrial ecotone and that they differ between host plant species. Furthermore, we hypothesize that (H2) these patterns are primarily driven by elevation-associated abiotic factors (soil bulk density, soil moisture, redox potential, soil pH, and soil salinity). Finally, we hypothesize that (H3) AMF colonization of plant roots increases with elevation along the marine-terrestrial ecotone as flooding stress decreases, and that (H4) AMF community composition varies along the ecotone in response to abiotic changes and host identity.

## Materials and methods

### Study site

**Fig. 1:**
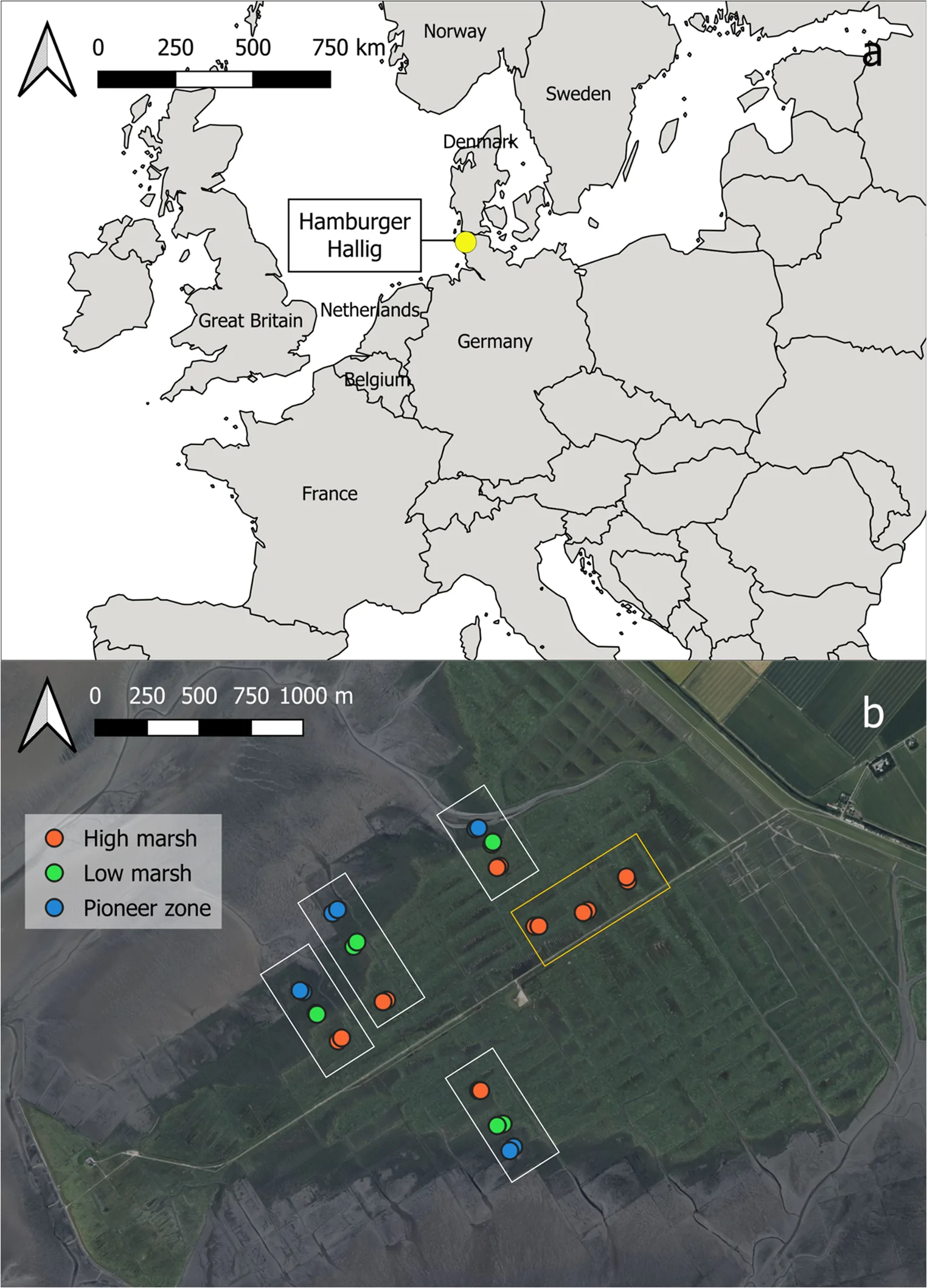
(**a**) Map of central Europe, showing the location of the study site “Hamburger Hallig” at the North Sea coast of Germany (yellow point). Map was taken from https://ec.europa.eu/eurostat/de/web/gisco/geodata/administrative-units/countries accessed on 13.11.2025 © EuroGeographics for the administrative boundaries. (**b**) Satellite image of Hamburger Hallig. Here, root and soil sampling was carried out in August 2022. Each dot marks a 1.5 × 1.5 m plot, in which soil samples were taken within stands of the two dominant plant species in each salt-marsh zone (color-coded). Some dots disappear behind each other on the map, because the plots were very close to each other. The white boxes show four transects covering the whole elevational gradient from marine-dominated pioneer zones to terrestrial-like high marshes, while the orange box shows six additional high marsh plots. Satellite image from Hamburger Hallig, Microsoft Bing Maps, accessed on 11.09.2025 © Microsoft Corporation

The study was conducted at the Hamburger Hallig (54°35’58’’N, 8°49’8’’E), Germany, a temperate salt marsh of approx. 3600 ha (Fig. 1). It is part of the Schleswig-Holstein Wadden Sea National Park and the Wadden Sea UNESCO World Heritage. The mean annual temperature between 1991 and 2021 was 9.6 °C, and the mean annual precipitation was 859 mm (Deutscher Wetterdienst, DWD). The tidal range at the site is approximately 3.0 m (Stock 2011).

### Study design and sampling

Across the Hamburger Hallig salt marshes, four transects were laid out (white boxes in Fig. 1b), each covering the three major vegetation zones from the lowest PIO zone, via LM zone at mid elevations, to terrestrial-like HM zone at the highest elevation. Along these transects, two plots of 1.5 × 1.5 m were randomly chosen in each zone. Within each plot, a soil block from beneath each of two selected plant species per zone were taken (PIO: *Salicornia europaea* agg. L., *Spartina anglica* C.E. Hubbard (syn.: *Sporobolus anglicus* P.M. Peterson & Saarela, naming follows Bortolus (2019)); LM: *Puccinellia maritima* (Huts.) Parl., *Atriplex portulacoides* L.; HM: *Elymus athericus* (Link) Kerg, *Festuca rubra* L. Throughout the whole following text, we refer to the plant species by their genus name. Additionally, we sampled another six plots to cover two different management regimes in the HM (ungrazed vs. moderately grazed by sheep (0.75 to 1.5 sheep per ha) from spring until autumn; ungrazed HM plots in white boxes, grazed HM plots in orange box in Fig. 1b). Here, we took from each plot a soil block of the two dominant plant species (*Elymus athericus*, *Festuca rubra*). During the analysis, we did not detect any statistically significant differences in RAF between grazed and ungrazed sites. We therefore decided to drop this comparison and incorporated the corresponding ‘grazed’ HM samples to the larger dataset covering the zonation gradient. This results in a total of *n* = 60 samples. We carried out sampling at the peak of the vegetation period in August 2022. Soil blocks were taken with a spade from the plots randomly, where the targeted plant species was growing in mono-specific stands, resulting in a soil sample size of 10 × 10 × 20 cm. The soil samples were placed into plastic bags and put into a fridge at 4 °C until further processing.

Soil bulk density, soil moisture, and redox potential were measured directly in each plot to characterize local environmental conditions. Soil bulk density was measured next to each soil block from a soil core with a volume of 100 cm^3^. Soil moisture content was measured with a hand-held meter (HH2, Delta-T Devices Ltd, Cambridge UK) and redox potential with a Pt-tipped redox electrode and an Ag/AgCl reference electrode (ORP, ecoTech, Bonn GER), both at 5 cm soil depth. Raw redox data were corrected to the potential of the standard hydrogen electrode to yield Eh (+ 207 mV). Eh data were not corrected for pH because a linear Eh–pH relationship in soils is questionable (Mansfeldt 2003).

### Sample processing (soil samples, root samples)

The soil samples collected in the fields were carefully washed to separate roots and bulk soil. The removed soil was preserved for measurements of pH and electrical conductivity (EC, proxy for salinity). For measuring pH, we powdered 5 g of dried soil (72 h at 60 °C), placed it into a 50 ml falcon tube, added 12.5 ml of distilled water, mixed it, let it rest overnight and measured with a pH Meter (Multi 9310 IDS, Xylem Analytics, Weilheim GER). For measurement of EC, we powdered 5 g of dried soil (72 h at 60 °C), placed it into a 50 ml falcon tube, added 25 ml of distilled water, mixed it, let it rest for 30 min and measured with a conductometer inoLab Cond7310 (Xylem Analytics, Weilheim GER).

The washed roots were homogenized and separated into two parts: 500–1000 mg of wet roots were placed into paper bags and dried at 60 °C for 72 h for DNA-based profiling of RAF communities. The remaining rest of the roots were placed into a 50 ml falcon tube and filled up with 35% ethanol for staining-based microscopic quantification of root colonization by AMF.

### Root staining

To quantify the AMF colonization in the roots, we followed the protocol of Ho-Plágaro et al. (2020) with slight modifications. Instead of the lactic acid solution, we used a mixture of lactic acid: glycerol: water (1:1:1 by volume) and we adapted the timing of placement of roots for each plant species in each solution to ensure optimal staining (Table 1).

**Table 1:** Plant species and the times (in minutes) for which roots were put in each solution for staining the AMF colonization structures, HCl: hydrochloric acid, KOH: potassium hydroxide, PIO: pioneer zone, LM: low marsh, HM: high marsh. Incubation in KOH (10%) and Trypan blue (0.05%) were carried out in a water bath at 80 °C, whereas incubations in HCl were carried out at room temperature

Plant species	Marsh zone	KOH [min]	HCl [min]	Trypan blue [min]
*Salicornia europaea*	PIO	15	3	20
*Spartina anglica*	PIO	20	3	20
*Puccinellia maritima*	LM	15	3	20
*Atriplex portulacoides*	LM	10	3	10
*Festuca rubra*	HM	25	3	10
*Elymus athericus*	HM	30	3	20

Thereafter, we placed 15 stained root segments of ~ 1.5 cm length per sample on a slide, prepared two slides per sample and followed the Magnified Intersections Method (McGonigle et al. 1990) for the quantification of AMF colonization. For the microscopy, we used the 40×−100× magnification of an Evident Scientific CX43RF microscope (T2 SN 3G43102, Nagano, Japan). For counting the AMF structures, each root segment was crossed five times by the eyepiece and in each intersection, we recorded presence/absence of AMF hyphae, arbuscules and vesicles. To calculate the proportion (%) of root length containing either hyphae, arbuscules or vesicles, we divided the recorded numbers by the total number of intersections of the two slides observed per sample and multiplied it with 100.

### RAF community profiling

For conducting the RAF community analyses, we milled the dried roots, weighed 20 mg per sample, and extracted the DNA using a commercial kit (DNeasy^®^ Plant Mini Kit, QIAGEN, Venlo, Netherlands), following the manufacturer´s recommendations.

For analysis of the RAF community and for the AMF community specifically, two PCR amplicons were generated from each sample, using primers targeting either fungal internal transcribed spacer (ITS2) region (Faghihinia et al. 2024) or part of 18S of AMF (Sun et al. 2025), respectively. Amplicons were prepared in technical triplicates using cycling conditions reported previously (see references above), mixed together per sample and purified using QIAquick PCR purification kit (QIAGEN, Venlo, Netherlands). Further, the amplicons from individual DNA samples were doubly indexed as described previously (Dudáš et al. 2022), mixed in equimolar concentrations and subjected to massively parallel amplicon sequencing. This was carried out on Illumina MiSeq (2 × 300 bp) platform at the Joint Genomic Facility at the University of Vienna. Demultiplexed reads were quality filtered (only sequences with Phred quality scores above or equal 30 per read and above or equal 7 per base were retained), paired and filtered to remove possible chimeras. Further, the sequencing data were processed as detailed in Bukovská et al. (2021). Briefly, ITS2 sequences were extracted from the amplicons, whereas from the 18S sequences, only the sequencing adaptors and primers were trimmed using the SEED2 software (Větrovský et al. 2018). Sequences were clustered at 97% similarity radius and reference (most abundant per cluster) sequences for each cluster identified using the SILVA (AMF sequences, Chuvochina et al. 2026) and UNITE (fungal ITS2 sequences, Abarenkov et al. 2024) databases. Further, non-target sequences (such as sequences identified as originating from plants and protists) were removed, and the remaining sequences resampled down to 5000 reads per sample for the fungal ITS2, and to 3000 reads per sample for the AMF sequences. Further, sequences were clustered again at 97% similarity and reference sequences identified using the EUKARYOME database (Tedersoo et al. 2024). Operational taxonomic units (OTUs; clusters within 97% similarity radius) were then merged according to the identity of phyla (ITS2 dataset) or AMF genera (18S dataset), while retaining information about their relative abundances.

### Statistical analyses

All statistical analyses were carried out in RStudio 2025.5.0.496 (RStudio Team 2025) based on R 4.5.0 (R Core Team, 2026). During the code development for the statistics in R, we used a generative AI assistant (perplexity.ai; accessed April - December 2025) to obtain suggestions for resolving minor coding errors and for improving code efficiency. All code was written, adapted and verified by the authors, and all analyses and interpretations were performed by the authors. For all graphs, we used the R package ggplot2 (Wickham 2016). The significance level was set to *p* < 0.05.

For the analysis of the sequencing data on community composition of RAF, we performed similar statistical analysis for the sequencing results of ITS2 amplicons and AMF amplicons (18S), while for the latter we only used the HM samples, as we had statistically too low numbers of positive samples from PIO and LM zones. All subsequent functions originate from the R package vegan (Oksanen et al. 2025), unless stated otherwise. We first determined the sufficiency of the sampling effort with a rarefaction curve analysis for both datasets using the function rarecurve, and excluded samples with very low counts (< 1000 reads for AMF and < 2000 reads for the ITS2 dataset) for further analysis. To perform a Principal Coordinate Analysis (PCoA), we calculated a rarefied Bray-Curtis distance matrix with the function avgdist, and used this for the PCoA with the function cmdscale from the R package stats (version 4.51). We performed a dispersion analysis of the AMF community with the function betadisper, followed by permutation test with the function permutest. To create the faceted bubble plots, we calculated relative abundance per fungal phylum (ITS2 dataset) or per genus (AMF dataset). To test for statistically significant differences in the composition of the RAF (ITS2 dataset) between the plant species of all zones, and AMF communities between the two plant species of the HM, we performed a PERMANOVA using the adonis2 function, followed by a pairwise post hoc test using the pairwise.adonis2 function from the R package pairwiseAdonis (Arbizu 2020). To test for the influence of the measured environmental variables (soil bulk density, soil moisture content, soil redox potential, soil pH, and soil salinity) on the fungal community composition, we performed a redundancy analysis (RDA) using the function rda, followed by a forward selection by the adjusted R^2^ and permutation tests using the function ordiR2step to find the best set of environmental predictors. For the assignments of functional guilds to fungal OTUs based on ITS2 sequencing data, we used the python-based FUNGuild tool (https://github.com/UMNFuN/FUNGuild) and followed the developers’ instructions (Nguyen et al. 2016). We parsed the taxonomic assignments at lowest possible taxonomic level, retaining only samples with > 2000 reads, and including guild assignments with confidence ranking of “highly probable” or “probable”. To minimize uncertainty in guild-level analysis, we aggregated guilds into broader trophic modes for subsequent community analysis.

To test for differences in AMF abundance in roots, measured through the colonized root length (%) between the salt marsh zones and the two plant species in each zone, we performed multivariate Kruskal-Wallis-Tests across the multiple variables representing colonization (hyphae, arbuscules and vesicles). Due to non-normality of the response variables, ANOVA approaches were not suitable and Kruskal-Wallis-Tests with the function kruskal_test from the R package stats were carried out (version 4.51). A post hoc dunn test with the Bonferroni p-adjustment-method using dunn_test from the package rstatix followed the Kruskal-Wallis-Test to test for pairwise differences in AMF root colonization between the salt marsh zones and plant species (Kassambara 2023).

## Results

**Fig. 2:**
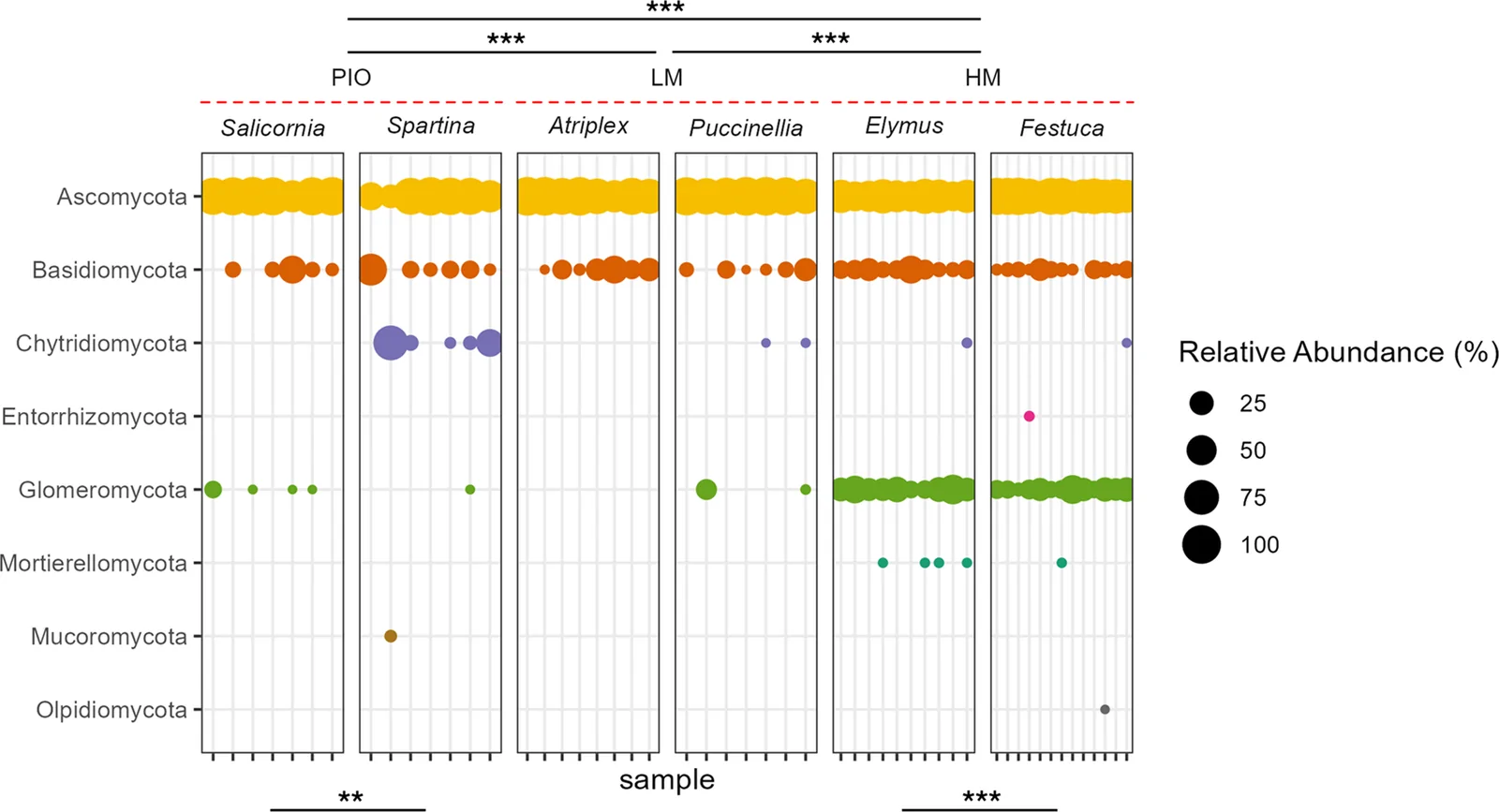
Community composition of RAF phyla based on ITS2 sequencing, displayed in the two dominant salt marsh plant species per salt marsh zone in the marine-terrestrial ecotone (PIO: pioneer zone, LM: low marsh, HM: high marsh). The x-axis represents individual samples, on the y-axis eight fungal phyla are displayed. Asterisks represent significant differences between the salt marsh zones (top) and between plant species within each zone (bottom) based on PERMANOVA followed by a permutation test with pseudo-*F* ratios (**: *p* ≤ 0.01, ***: *p* ≤ 0.001)

Overall, the RAF community in the salt marsh plants was dominated by Ascomycota (77.23 ± 17.91%), followed by Glomeromycota (10.9 ± 12.66%) and Basidiomycota (10 ± 12.86%). Ascomycota and Basidiomycota were present in nearly equal proportions across the salt marsh zones of the marine-terrestrial ecotone (Ascomycota: PIO 76.11 ± 27.78%, LM 88.33 ± 11.48%, HM; 70.63 ± 12.98%; Basidiomycota: PIO 13.6 ± 21.13%, LM 10.37 ± 11.68%, HM 8.29 ± 9.12%, Fig. 2). The observed significant differences in RAF community composition among the salt marsh zones (PERMANOVA, *p* = 0.001, R² = 0.22878; Fig. 2) were largely shaped by differences in the occurrence of other fungal phyla. We found significant differences of RAF community structure between the plant species in both PIO and for HM, but not in LM (PERMANOVA, *p* = 0.001, R² = 0.35046; Table 2).

**Table 2:** Statistical results from a permutation test with pseudo-*F* ratios following a PERMANOVA testing the difference between the three salt marsh zones in the marine-terrestrial ecotone and the two plant species in each salt marsh zone, displaying the calculated R^2^, the F value and the p value (*p* < 0.05 shown in bold)

Factor	*R* ^2^	F value	*p* value
PIO x LM	0.11	3.4	**0.001**
LM x HM	0.16	7.1	**0.001**
PIO x HM	0.25	11.5	**0.001**
PIO: *Spartina* x *Salicornia*	0.17	2.5	**0.004**
LM: *Atriplex* x *Puccinellia*	0.10	1.4	0.072
HM: *Festuca* x *Elymus*	0.21	5.6	**0.001**

**Fig. 3:**
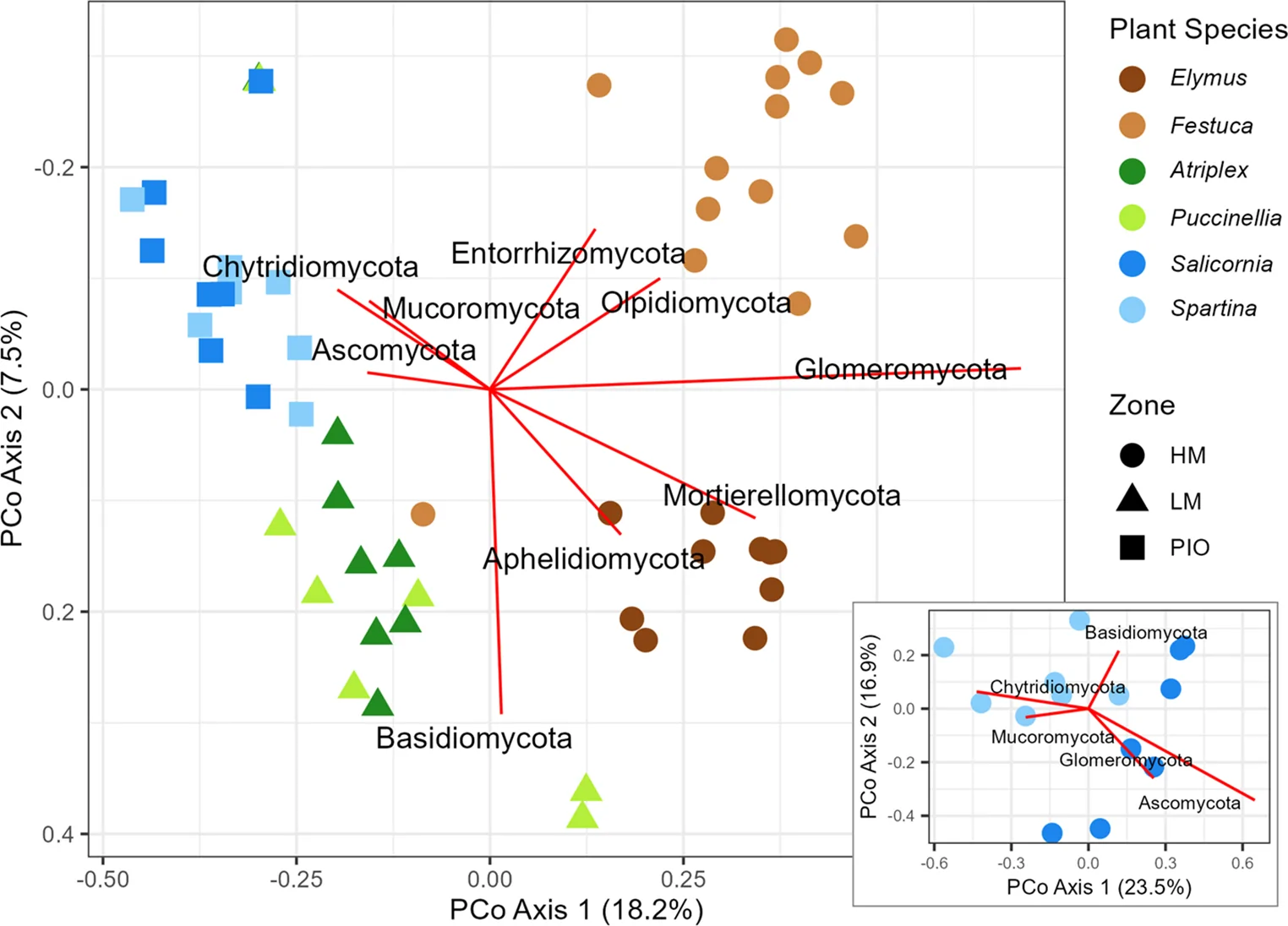
Principal coordinates analysis displaying RAF community composition based on ITS2 sequencing between axis 1 and 2 on large panel with the whole dataset. The small panel shows the PCoA just for the PIO dataset (inset). Samples are coded according to salt marsh zone in the marine-terrestrial ecotone (PIO: pioneer zone, LM: low marsh, HM: high marsh) and according to plant species (*Spartina anglica*, *Salicornia europaea*, *Puccinellia maritima*, *Atriplex portulacoides*, *Festuca rubra*, *Elymus athericus*)

Based on amplicon sequencing, Chytridiomycota were abundant in PIO zone, particularly in *Spartina* roots (26.69% ± 42.18%), but not in *Salicornia.* They were almost absent in LM (0.01 ± 0.02%) and HM zones (0.01 ± 0.04%). In the PIO, Mucoromycota was found in *Spartina* (0.39 ± 0.68%), while we also detected low amounts of Glomeromycota in *Salicornia* (0.85 ± 2.55%). Mortierellomycota occurred at low abundance in both HM species (*Elymus*: 0.04% ± 0.05%, *Festuca*: 0.01 ± 0.03%), while Entorrhizomycota and Olpidiomycota were restricted to *Festuca* in the HM (Entorrhizomycota: 0.02 ± 0.06%, Olpidiomycota: 0 ± 0.01%). We found very low amplicon abundances of Glomeromycota both in PIO (0.85% ± 2.55%) and LM zones (1.1% ± 4.05%) compared to the high relative abundance in HM (19.75% ± 11.77%) (Fig. 2). The contrast between the fungal community in the PIO and the high abundance of Glomeromycota in the HM explained 18.2% of the variation in RAF community structures, as indicated by the parallel arrows to the x-axis in the PCoA plot (Fig. 3). Additionally, the PCoA based on Bray-Curtis revealed distinct RAF communities between the two HM plant species *Elymus* and *Festuca* (Fig. 3). In contrast, the RAF communities of the different host species (*Spartina* and *Salicornia*) within the PIO zone showed considerable overlap in the two-dimensional ordination space (Fig. 3). However, PERMANOVA still detected a significant, yet more subtle host plant effect (R^2^ = 0.17, *p* = 0.004), which becomes visually detectable upon PCoA for just the PIO samples (Fig. 3 - inset). The RAF community of the LM showed considerable overlap in the PCoA (Fig. 3) and no statistical differences in PERMANOVA (Table 2), and thus more similarity between the two respective plant species (*Atriplex* and *Puccinellia*).

**Fig. 4:**
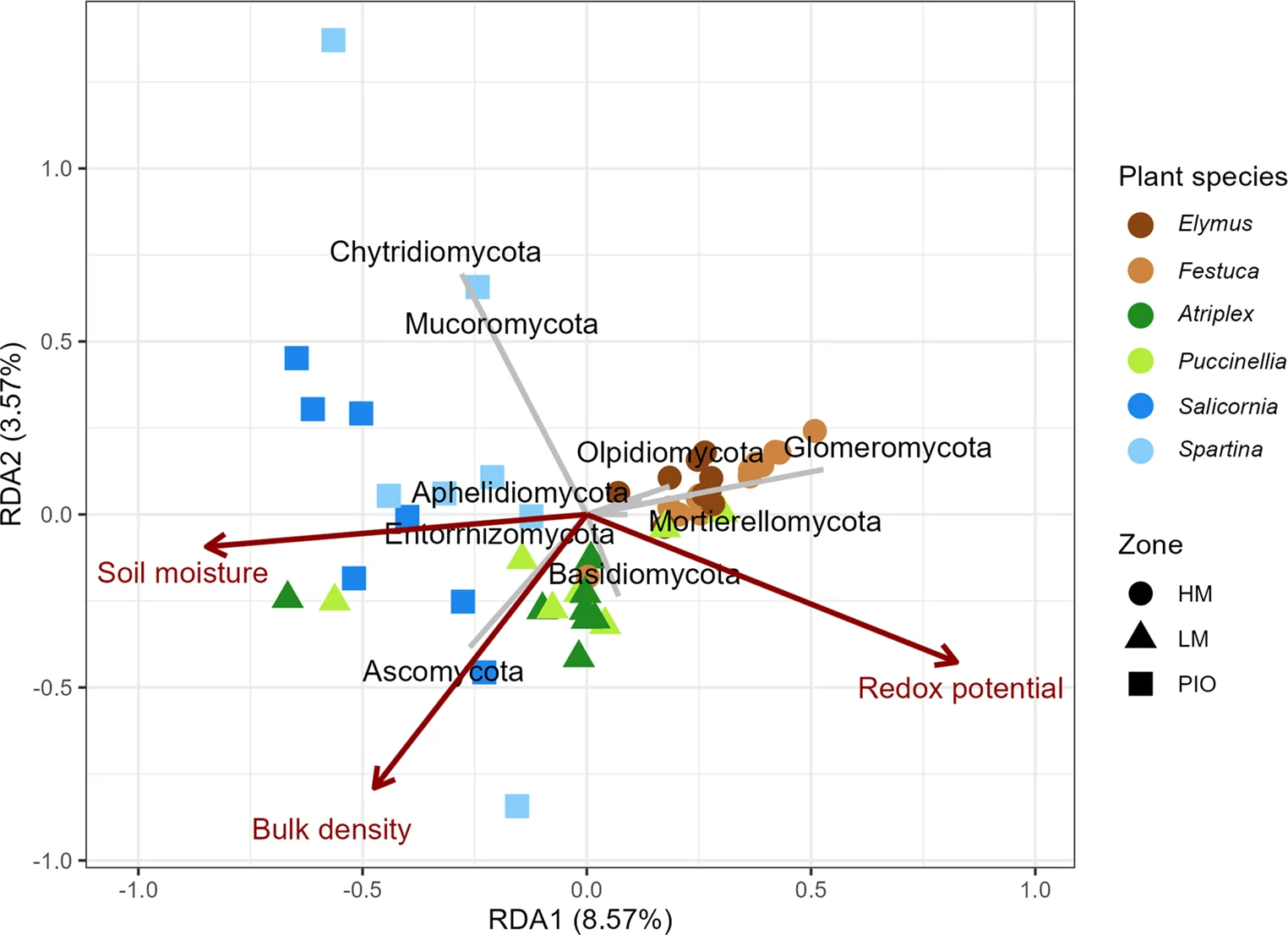
RDA of RAF phyla based on ITS2 sequencing of the plant species per salt marsh zone in the marine-terrestrial ecotone with the three most important environmental variables displayed as dark red arrows (PIO: pioneer zone, *Salicornia europaea*, *Spartina anglica*; LM: low marsh, *Atriplex portulaciodes*, *Puccinellia martitima*; HM: high marsh, *Elymus athericus*, *Festuca rubra*)

Based on RDA forward selection, we identified the redox potential (~ proxy for oxygen availability in the soil) as the most important environmental factor associated with the RAF community composition (RDA permutation test, *p* = 0.004, R^2^_adj_ = 0.047). A reduced RDA model containing the three strongest environmental predictors (redox potential, soil moisture, and bulk density) significantly explained the community composition (RDA permutation test, *p* = 0.002, Supporting Information Table S1). Within this model, the first two axes captured 8.62% (RDA1) and 3.57% (RDA2) of the total variation, reflecting biochemical and physical gradients shaping the community (Fig. 4, Supporting Information Table S1). Along the gradients, soil moisture points towards the samples originating from the PIO, the wettest of the three zones (Supporting Information Fig. S1). Soil redox potential is also almost parallel to RDA axis 1 and points in the opposite direction of soil moisture, explaining most of the variance captured by this axis. It ranges from very low values in the marine-dominated PIO (−100 mV), which associates with Ascomycota, Chytridiomycota and Mucoromycota, to high redox values in the terrestrial-like HM (+ 450 mV), where fungal communities tightly cluster in association with Glomeromycota in roots of *Festuca* and *Elymus.*

**Fig. 5:**
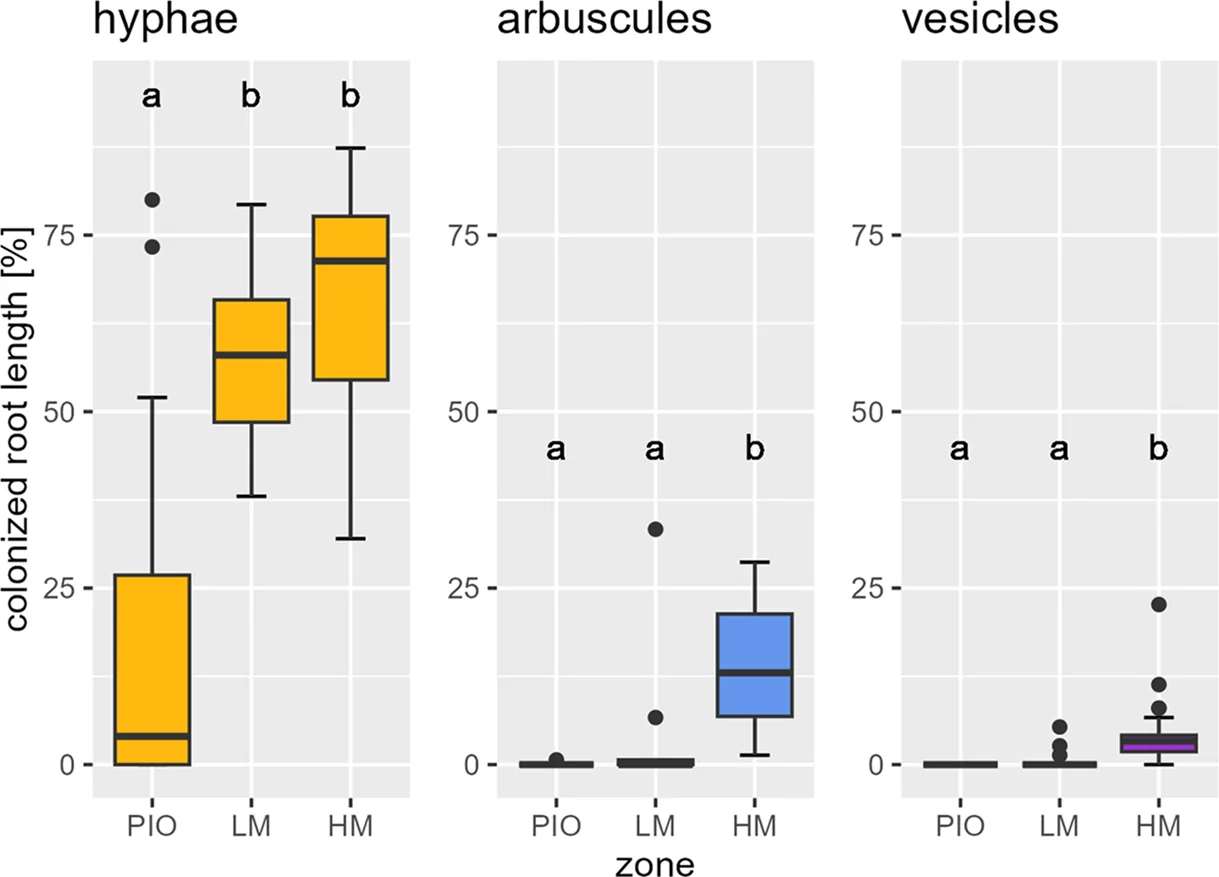
Arbuscular mycorrhizal colonization (% of root length) by hyphae, arbuscules, and vesicles as determined by microscopy per salt marsh zone in the marine-terrestrial ecotone (PIO: pioneer zone, LM: low marsh, HM: high marsh) in the two analyzed plant species per salt marsh zone. Small letters indicate significant differences (*p* ≤ 0.05%), based on Kruskal-Wallis-Tests and Post-Hoc Dunn tests

Root colonization with AMF in salt marsh plants showed a significant increase from the marine-like PIO to the terrestrial-like HM (multkw; hyphal colonization: *p* < 0.001, arbuscular colonization: *p* < 0.001, vesicular colonization: *p* < 0.001). The hyphal colonization was significantly lower in PIO (18.37 ± 27.03%) than in LM (57.79 ± 12.53%) and HM (66.36 ± 15.23%) (Fig. 5; Post-Hoc Dunn Test; PIO vs. LM: *p* = 0.0111; PIO vs. HM: *p* < 0.001). The pattern of arbuscular and vesicular colonization differed slightly from the hyphal colonization: In the lower zones, arbuscules (PIO: 0.04 ± 0.17%; LM: 2.63 ± 8.35%) and vesicles (PIO: 0%; LM: 0.58 ± 1.46%) were almost non-detectable or even absent. In the HM, we detected a significantly higher arbuscular (14.05 ± 8.61%) and vesicular (4.19 ± 4.5%) colonization of the roots (arbuscules: Post-Hoc Dunn Test; PIO vs. HM: < 0.001; LM vs. HM: *p* < 0.001; vesicles: Post-Hoc Dunn Test; PIO vs. HM: < 0.001; LM vs. HM: < 0.001). In general, we detected no significant differences in AMF abundance between the two analyzed plant species per zone (Supporting Information Fig. S2).

**Fig. 6:**
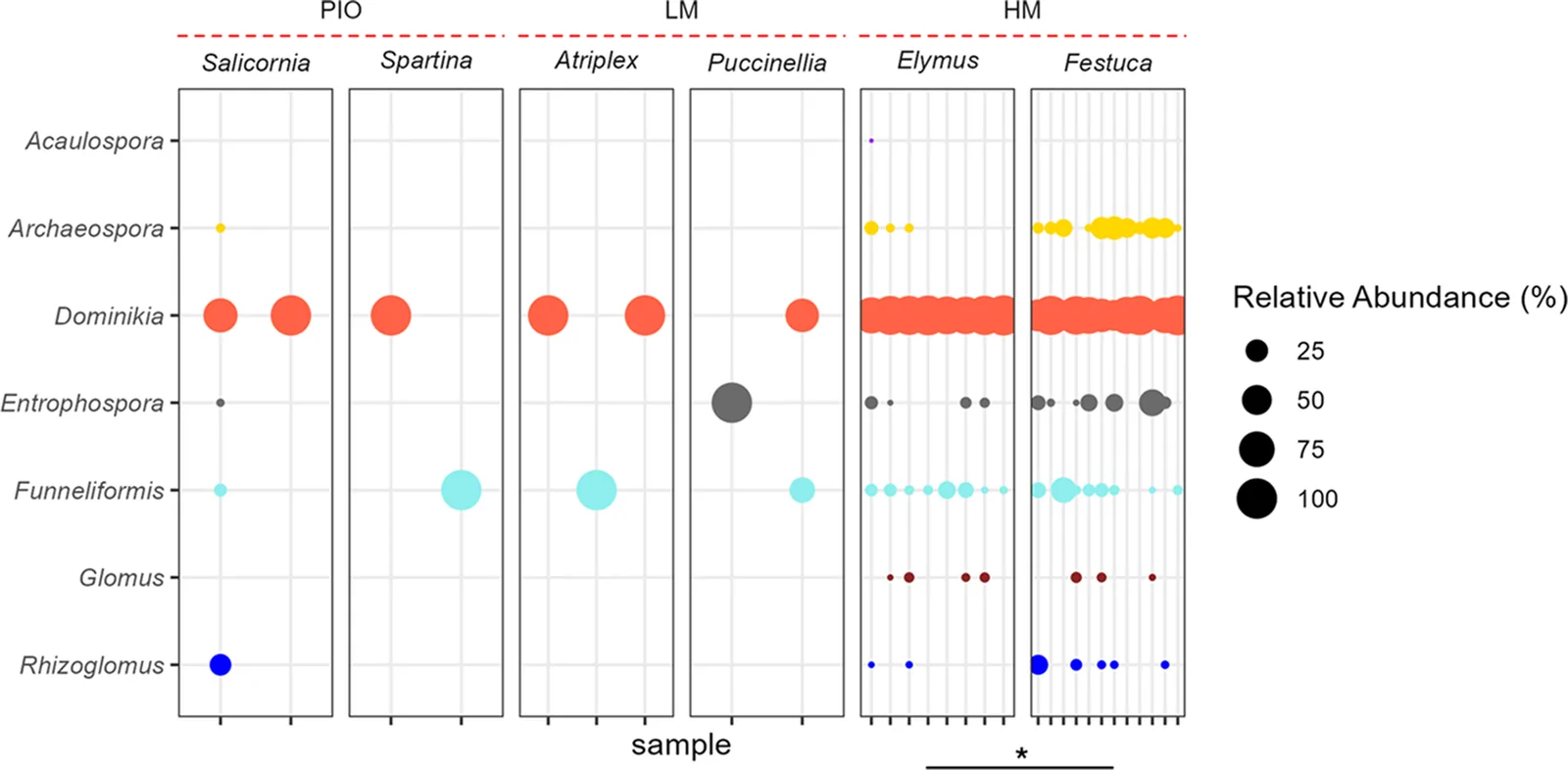
The community composition of AMF genera based on 18S sequencing, displayed for each salt marsh zone in the marine-terrestrial ecotone (PIO: pioneer zone, LM: low marsh, HM: high marsh) from the two analyzed plant species per salt marsh zone (*Spartina anglica*, *Salicornia europaea*, *Puccinellia maritima*, *Atriplex portulacoides*, *Festuca rubra*, and *Elymus athericus*). The x-axis represents samples. Asterisks below the plot represent significant differences within plant species in the terrestrial-like HM based on PERMANOVA followed by a permutation test with pseudo-*F* ratios (*: *p* ≤ 0.05)

**Fig. 7:**
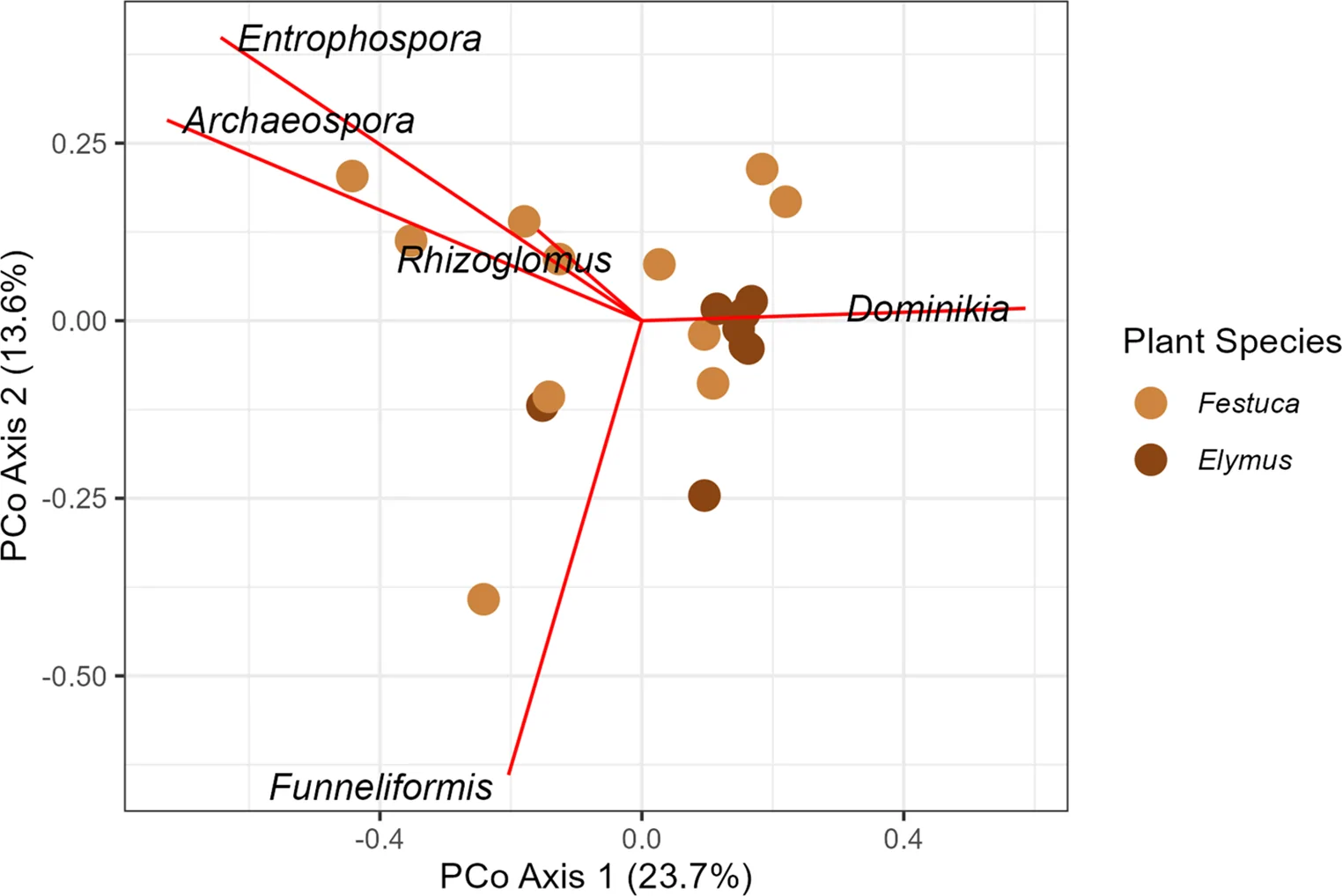
Principal coordinates analysis displaying AMF community composition based on 18S sequencing from two salt marsh plant species (*Elymus athericus*, *Festuca rubra*) from the terrestrial-like high marsh zone

Most detected AMF were thus present only in the terrestrial-like HM, whereas we were able to amplify positively only few samples from marine-dominated PIO and LM at mid elevation (Fig. 6). The AMF genus *Dominikia* dominated the AMF community in all salt marsh zones (PIO: 67.1 ± 47.15%; LM: 53.11 ± 50.48%; HM: 80.07 ± 17.39%). In the terrestrial-like HM, other genera such as *Funneliformis* and *Entrophospora* were also abundant in *Festuca* (*Funneliformis*: 5.39 ± 9.7%; *Entrophospora*: 6.73 ± 11.28%) and *Elymus* (*Funneliformis*: 5.18 ± 4.54%; *Entrophospora*: 1.64 ± 2.36%). Despite this overlap in dominant taxa across the two plant species, the AMF communities associated with *Elymus* and *Festuca* differed significantly in overall composition (PERMANOVA, *p* = 0.033, R² = 0.09033). The AMF communities associated with *Elymus* were significantly less dispersed than those associated with *Festuca* (betadisper, permutation test, *p* = 0.007), indicating higher compositional homogeneity of AMF in *Elymus* (Fig. 7). These communities were also more strongly associated with the genus *Dominikia*, as shown by the proximity of the *Dominikia* vector to PCoA axis 1, which explains 23.9% of the total variation (PCoA, Fig. 7). In contrast, the AMF community associated with *Festuca* was more heterogeneous, as shown by the wider spread of *Festuca*-associated samples in the PCoA plot (Fig. 7). While *Dominikia* was also frequently detected in roots of *Festuca* roots (73.4 ± 19.23%), the community composition included a broader range of taxa such as *Archaeospora* (11.59 ± 10%), *Rhizoglomus* (2.24 ± 5.23%), and *Glomus* (0.54 ± 1.14%), indicating greater taxonomic diversity as compared to *Elymus.*

## Discussion

This study presents a thorough analysis of how host plant identity and a strong environmental gradient in the marine-terrestrial ecotone affect RAF communities based on ITS2 and 18S sequencing and AMF root colonization assessed microscopically. We show that (H1) RAF communities shift significantly along the elevational gradient from the marine-dominated pioneer zone to the terrestrial-like high marsh and that RAF communities differ between host species within salt marsh zones, with the exception of the LM. We further found that, (H2) these shifts are mainly associated with changes in redox conditions, and that (H3) AMF colonization of roots significantly increases with elevation, and (H4) AMF community in the HM is significantly affected host identity. Whereas the effects of abiotic soil factors remained insignificant in the latter analysis, this is something that is not very surprising as soil conditions were generally similar among samples collected from the HM zone only. Whether the AMF community was also affected by abiotic factors in the marine-terrestrial ecotone could not be rigorously tested here as AMF were almost absent from the DNA amplicons of samples obtained from the PIO and LM zones. These results collectively demonstrate how environmental factors including soil conditions and plant host identity correlate with RAF communities within salt marshes of the marine-terrestrial ecotone.

Along the elevational gradient of the ecotone, there were significant differences in the abundance and community composition of RAF. Occurrence increased from the often-flooded and marine-dominated PIO to the terrestrial-like HM, and the RAF communities were clearly zone-specific. Chytridiomycota and Mucoromycota, two fungal groups that are known to tolerate low oxygen availability, were mostly present only in the communities of the marine-dominated PIO (Ortega-Arbulú et al. 2019; Ogola et al. 2025). Besides, there is ample evidence for occurrence of these phyla in marine environments (Naranjo-Ortiz and Gabaldón 2019). Among the soil variables measured in this study, soil redox potential stood out as the main factor correlating with the composition of RAF communities along the elevational gradient. In the frequently flooded and marine-dominated PIO, low redox potential conditions likely limit the occurrence of fungi in roots due to reduced oxygen availability, which in turn affects their growth, penetration, and metabolic activity, as previously shown for AMF (Maček et al. 2011; Maček 2017). Similar redox-associated shifts in microbial communities have been reported for other wetland soils (Lüdemann et al. 2000; Edlund et al. 2008; Lipson et al. 2015; Boie et al. 2025). But the results of our study go even further and show that these abiotic conditions in the soil also significantly influence fungal communities developing inside roots, not only in soils. Specifically, we observed a pronounced transition from saprotrophic or pathogenic fungal communities dominating in marine-dominated PIO and LM (Chytridiomycota, Mucoromycota, Ascomycota and Basidimycota) to symbiotrophic fungi (Glomeromycota) in the terrestrial-like HM (see also FUNGuild results in Supporting Information Fig. S3). The restricted occurrence of AMF at lower elevation (PIO and LM) and much more frequent occurrence at higher elevation (HM), suggests that oxygen availability and flooding stress strongly affect the environmental range (realized niche) of those obligate symbionts, although we cannot rule out at this stage that the plant hosts of PIO and LM themselves actively suppress the symbiosis. This is partly due to correlative nature of our experimental evidence and also due to strong vegetation zonation within this particular ecotone, where no single plant species inhabits all elevational zones – thus unbiased interpretations of field data remain difficult. Overall, the functional turnover of fungal guilds along the elevational gradient suggests fundamental ecological consequences for salt marsh systems: The transition from pathogenic/saprotrophic pressure in the lower zones to mutualistic AMF symbiosis in the HM might directly modulate host plant performance and can lead from costly stress-mitigation in the PIO/LM zones to enhanced nutrient acquisition and biomass production for plants in the HM zone due to local RAF communities. Yet, future work should experimentally and directly link these observed guild transitions (changes along the gradient) to direct measurements of host fitness under uniform soil conditions, to disentangle causes and consequences of the observed phenomena.

Moreover, within salt marsh zones, host plant identity apparently strongly influenced RAF communities. Significant host-specific differences were detected in the PIO (*Salicornia* vs. *Spartina*) and HM zones (*Elymus* vs. *Festuca*), whereas we detected no differences between the two plant species in the LM zone (*Atriplex* vs. *Puccinellia*). This fluctuating relative importance of environmental vs. host control along the elevational gradient seems to require more nuanced perspective for explaining assembly of the RAF communities. In the highly stressed PIO, host identity apparently has a strong effect on the RAF communities, even though this subtle variation is visually nested within the dominant macro-environmental trends of the overall PCoA (Fig. 3). The absence of host specificity in the LM plants may reflect non-exclusive mechanisms. First, LM vegetation exhibits higher small-scale plant diversity compared to both PIO and HM, where the plant species often occur in monospecific stands (Suchrow et al. 2015). Increased local diversity may actually dilute host-selective effects by increasing fungal sharing through the soil among neighboring plants, as observed in other systems (Li et al. 2021). Second, LM roots were dominated by Ascomycota and Basidiomycota, globally distributed fungal phyla (Egidi et al. 2019; He et al. 2022), which may exhibit broader host ranges and therefore weaker host specificity. Third, the high salinity combined with occasional droughts in the LM might be too intense for many fungi. Or fourth, suppression of PCR efficiency may have prevented our primers from efficient fungal DNA amplification from LM samples, a known phenomenon for plants cross-reactive with the fungi-targeting primers or producing high levels of secondary metabolites acting as PCR inhibitors (Viotti et al. 2024). In fact, the primers appear to have predominantly amplified host DNA for the LM samples due to cross-amplification, consequently leading to apparent lack of difference in RAF communities between the two LM plant species. The re-emergence of the host-control in the HM suggest that while broad-scale abiotic filtering defines the potential species pool across the entire gradient, the host plants might still to a large extent determine realized associations from within that pool (Davison et al. 2016; Deng et al. 2026). Our findings suggest that the secondary, host mediated environmental filter varies depending on the specific environmental stress in each zone. While in the PIO, specific host traits might be critical to buffer against extreme conditions, the different host plants have developed highly distinct strategies to survive under extremely (but otherwise rather constant) stressful conditions, thereby creating their own respective niches (Metzing 2023). In contrast, in the HM, host identity seems to prevail in dictating community structure in absence of strong abiotic constraints. However, since specific plant traits, like aerenchyma development, radial oxygen loss, and exudation patterns were not explicitly quantified in our study, mechanistic understanding of the contribution of the different environmental drivers to the RAF communities still remains limited. To improve it, targeted experimentation to disentangle contribution of the different environmental factors to RAF community assembly will need to be developed in the future, using hypotheses generated here.

Consistent with previous microscopy-based studies (e.g.: Rozema et al. 1986; Hildebrandt et al. 2001; Druva-Lusite and Ievinsh 2010) and sequencing-based analyses (Alzahrani 2017; Zhou et al. 2022; Ma et al. 2024), AMF colonization in salt marsh roots increased significantly from the marine-dominated PIO and LM at mid elevations toward terrestrial like HM. The observations of near absence of AMF colonization in often flooded PIO and LM zones, one of the strongest effects observed here, indicates that water-logged soils and low redox potential constrains the formation of this obligate symbiosis. Therefore, ecological exclusion rather than solely methodological bias is probably the cause of limited AMF sequence recovery in PCR amplicons outside the HM samples. In frequently flooded marine-dominated PIO, environmental stress did possibly prevent AMF colonization of host roots or plant hosts may have downregulated the development/maintenance of the symbiosis under conditions of high nutrient availability and low oxygen, conditions prevalent in the lower zones of salt marshes at the North Sea coast (Mueller et al. 2020). At higher elevations in terrestrial-like HM, in contrast, reduced inundation and increased seasonal drought might enhance the functional importance of AMF for the plants, resulting in their higher observed abundance. Mutualistic interactions namely can benefit plants increasingly under moderate stress conditions (Lekberg et al. 2018). AMF associations with salt marsh plants may improve nutrient acquisitions, boost stress tolerance, and increase pathogenic resistance (Farrer et al. 2022; Wang et al. 2022; Lumibao et al. 2024) potentially explaining their strong prevalence in the HM, although mechanistic proof for these claims is still missing.

In contrast to earlier studies reporting *Glomus* dominance in salt marshes (Hildebrandt et al. 2001; Wilde et al. 2009; Guo and Gong 2014; Guan et al. 2020; Tsiknia et al. 2021; Ma et al. 2024), *Dominikia* was the most abundant AMF genus in this study, followed by *Funneliformis*, *Archaeospora*, and *Entrophospora*. Given that *Dominikia* is a recently described genus (Błaszkowski et al. 2021), its apparent underrepresentation in earlier studies may reflect limitations in molecular markers and database coverage (Tedersoo et al. 2024). Additionally, many taxa previously treated as *Glomus* are now placed in *Dominikia*, so the apparent shift from *Glomus* to *Dominikia* dominance likely reflects taxonomic revisions, rather than genuine difference in community compositions across studies (Błaszkowski et al. 2021).

Because AMF were mainly restricted to terrestrial-like HM in the marine-terrestrial ecotone, the community analysis focused on this zone, where colonization and sequencing depth allowed robust comparisons. Here, we observed that host identity structured AMF communities more strongly than abiotic soil factors, as no large differences in environmental parameters were detected between *Elymus* and *Festuca* microsites (see Supporting Information Fig. S1). Interestingly, though, *Elymus* roots hosted relatively homogenous AMF communities, clearly dominated by *Dominikia*, which suggests a more selective partnership of the plant with this AMF genus. In contrast, the AMF community in *Festuca* roots included *Rhizoglomus* and *Archaeospora*, and therefore supported a richer fungal community, hinting at a broader symbiotic strategy that increases functional flexibility of the host (Powell and Rillig 2018). With this study in the marine-terrestrial ecotone, our results suggest that once environmental stress is sufficiently reduced to permit symbiosis, establishment/maintenance within the roots, plants probably benefit more from the symbiosis than it would cost them. Besides, host traits appear to determine which AMF taxa successfully establishes within roots, although this conclusion is only based of comparison of two plants here and thus does not provide a very strong experimental proof as such – but is consistent with previous research (e.g., Jansa et al. 2002).

The findings from our study suggest that the capacity of salt marsh plants to associate with RAF (including the generally plant-beneficial AMF) is strongly determined by regular flooding stress and resulting soil conditions. With climate change-induced sea-level rise and changing inundation regimes, altering soil conditions including redox potential and salinity will likely modify root colonization by the AMF. If flooding frequency increases, reduced AMF colonization may impact plant nutrient acquisition and stress tolerance, potentially altering plant species distributions. Conversely, shifts towards drier conditions under rising temperatures and changes in precipitation patterns may enhance the ecological importance of host-specific RAF interactions. Future research should therefore experimentally manipulate flooding, redox and drought conditions to directly test linkages between environmental stress and root colonization levels and dynamics.

## Supplementary Information

Below is the link to the electronic supplementary material.

ESM 1 (DOCX 1.08 MB)

## Data Availability

Raw data and RScripts are available at [‘UHH’] https://doi.org/10.25592/uhhfdm.18492.Raw sequencing data were deposited in NCBI sequence read archive (SRA) database under the accession number PRJNA1390452.
