# Supplementary material for: Soil redox potential and plant species identity likely affect root-associated fungi in salt marshes
Source: Mycorrhiza. 2026 Sep 24;36(5):69. doi: 10.1007/s00572-026-01310-2 (PMC13601159; doi:10.1007/s00572-026-01310-2)
Supplement: Supplementary file 1 [file 572_2026_1310_MOESM1_ESM.docx]

**Soil redox potential and plant species identity likely affect root-associated fungi in salt marshes**

Andrea-Carolin Menzel^1#^, Lina Kleemann^1^, Petra Bukovská^2^, Jason N. Woodhouse^3,4^, Peter Mueller^5^, Kai Jensen^1^, Jan Jansa^2^

^#^corresponding author: [andrea-carolin.menzel@uni-hamburg.de](mailto:andrea-carolin.menzel@uni-hamburg.de)

^1^Institute of Plant Science and Microbiology, University of Hamburg, 22609 Hamburg, Germany

^2^Institute of Microbiology, Czech Academy of Sciences, 14200 Prague, Czech Republic

^3^Department of Biology, University of Hamburg, 20148 Hamburg, Germany

^4^Leibniz-Institute of Freshwater Ecology and Inland Fisheries, 16775 Stechlin, Germany

^5^Institute for Environmental Sciences, RPTU Kaiserslautern-Landau, 76829 Landau, Germany

**Figure S1**: Measured environmental factors in the soil block samples taken underneath the two dominant salt marsh plant species (*Spartina* *anglica*, *Salicornia europaea*, *Puccinellia* *maritima*, *Atriplex* *portulacoides*, *Festuca rubra*, *Elymus athericus*) from each zone (PIO: pioneer zone, LM: low marsh, HM: high marsh). Small letters indicate significant differences (p ≤ 0.05 %), based on ANOVAs and TukeyHSD tests. If no small letters are displayed, we did not find significant differences.

**Figure S2**: Arbuscular mycorrhizal colonization (% of root length) by hyphae, arbuscules, and vesicles as determined by microscopy per salt marsh zone in the marine-terrestrial ecotone (PIO: pioneer zone, LM: low marsh, HM: high marsh) in the two analyzed plant species per salt marsh zone. Small letters indicate significant differences (p ≤ 0.05 %), based on Kruskal-Wallis-Tests and Post-Hoc Dunn tests.

**Figure S3**: Relative abundance of sequencing reads per sample (x-axis) based on ITS2 sequencing and classifications of FUNGuild displayed in the two dominant salt marsh plant species (*Spartina* *anglica*, *Salicornia europaea*, *Puccinellia* *maritima*, *Atriplex* *portulacoides*, *Festuca rubra*, *Elymus athericus*) per zone (PIO: pioneer zone, LM: low marsh, HM: high marsh). The x-axis represents individual samples, the y-axis are the eight fungal trophic modes.

**Table S1**: Results of RDA models after forward selection, which is based on R^2^ and p-value (p < 0.05), testing which measured environmental variable(s) explain best the root-associated fungal community along the elevational gradient in North Sea salt marshes.

| Model Variables | | Redox potential | Redox potential, soil moisture, bulk density |
| --- | --- | --- | --- |
| Inertia  Proportion | Total  Constrained  Unconstrained  Total  Constrained  Unconstrained | 0.17  0.012  0.17  1.00  0.07  0.93 | 0.19  0.02  0.16  1.00  0.13  0.87 |
| Permutation Test | F value  P value | 3.53  0.004 | 2.41  0.002 |
| Adjusted R^2^ |  | 0.05 | 0.08 |
| RDA1  PC1/RDA2 | Eigenvalue  Proportion explained  Cumulative Proportion  Eigenvalue  Proportion explained  Cumulative Proportion | 0.01  0.07  0.07  0.42  0.23  0.29 | 0.02  0.09  0.09  0.01  0.04  0.12 |
